# Treatment duration with immune-based therapies in Cancer: an enigma

**DOI:** 10.1186/s40425-018-0465-0

**Published:** 2018-12-05

**Authors:** Shanta Bantia, Nirmal Choradia

**Affiliations:** 1 Nitor Therapeutics, 689, Highland Lakes Cove, Birmingham, AL-35242 USA; 2 Palo Alto Veterans Affairs Hospital, 3801 Miranda Ave, Palo Alto, CA 94304 USA

**Keywords:** Immunotherapy, Immune exhaustion, Treatment duration

## Abstract

Unlike chemotherapy treatments that target the tumor itself (rather nonspecifically), immune-based therapies attempt to harness the power of an individual patient’s immune system to combat cancer. Similar to chemotherapeutic agents, the dosage and Administration section of labeling for all five currently approved PD-1/PD-L1 inhibitors (immunotherapy) recommends duration of treatment until disease progression or unacceptable toxicity. Overactivation or constitutive activation of the immune system with immune based therapies can lead to T-cell exhaustion and activation induced cell death (AICD) in T- and B-cells. Examples of immune exhaustion and T-cell depletion is noted in preclinical and clinical studies. Overactivation or constitutive activation leading to Immune exhaustion is a real phenomenon and of profound concern as immune cells are the true arsenal for control of the tumor growth. Designing trials rigorously to address the optimum treatment duration with immune based therapies is critical. By addressing this concern now, not only we may improve patient outcomes, but also gather a deeper understanding of the role and mechanisms of the immune system in the control of tumor growth.

Chemotherapy and immune-based therapies provide antitumor effects through completely different mechanisms. Chemotherapeutic agents are cytotoxic in that they directly inhibit basic cellular mechanisms, killing both malignant and nonmalignant cells (hopefully with a preference for malignant cells), while immune based therapies wake-up the host immune system to recognize malignant cells and eliminate them.

While there is a burgeoning excitement surrounding development of immune based therapies for the treatment of cancer, the optimal duration for these therapies need to be explored with equal fervor. Dosing for chemotherapy has been determined over years through large-scale prospective randomized trials to pinpoint the dose which maximizes therapeutic effect while minimizing side effects. Also, due to the mechanism of chemotherapeutic action, the duration of treatment with these agents is generally until disease progression or patient intolerance. However, experience with immune based therapies is limited, with current dosing and duration guidelines based primarily on initial trials required for approval of the agents. Since immune based therapies work by activating the body’s own immune system, there is concern that **overactivation or constitutive activation of the immune system may lead to immune exhaustion and depletion of effector T-cells thereby causing decreased anti-tumor effects and possible allowing for tumor progression**.

Similar to chemotherapeutic agents, the Dosage and Administration section of labeling for all five currently approved PD-1/PD-L1 inhibitors recommends duration of treatment until disease progression or unacceptable toxicity. However, since immune based therapies work with a completely different mechanism compared to chemotherapy, using the same therapy duration may not be the optimal approach.

In exploring treatment duration with immune based therapies, we need to answer the following: (1) does indefinite treatment with immune based therapies exhaust the immune system counteracting its own mechanism of action leading to tumor progression and (2) how can clinical trials be designed to identify the optimal duration of immune-based therapy that prevents immune cell exhaustion but supports anti-tumor immunity.

## Overactivation of the immune system

Overactivation or constitutive activation of the immune system can lead to T-cell exhaustion and activation induced cell death (AICD) in T- and B-cells. Clinical examples of the detrimental effects of immune exhaustion have been studied in a number of other diseases including sepsis and chronic viral infections, where constitutive activation of the immune system eventually leads to immunosuppression through similar mechanisms to those described below [1–3].

AICD is programmed cell death in activated T cells caused by the interaction of Fas receptors (Fas, CD95) and Fas ligands (FasL, CD95 ligand) [4]. Both activated T-cells and B-cells express Fas and undergo clonal deletion by the AICD mechanism. Activated T-cells that express both Fas and FasL may be killed by themselves or by each other. FAS/FASL death-signaling pathway is induced during HIV disease and contributes significantly to viral pathogenesis and depletion of T-cells. Although the tumor cells express high levels of FAS, the role of this signaling pathway in eliminating T-cells in the tumor microenvironment is not clear.

Exhausted T-cells in cancer express high levels of inhibitory receptors, including PD-1, CTLA-4, TIM-3, LAG-3, BTLA and TIGIT, as well as show impaired effector cytokine production such as IL-2, TNF-α, IFN-γ and GzmB and are essentially ineffective in eliminating malignant cells. Deeply exhausted and terminally differentiated T-cells can also more frequently undergo AICD and apoptosis. A few examples of immune exhaustion in preclinical and clinical studies are listed below.

Some of the combination studies performed with checkpoint modulators have demonstrated T-cell exhaustion and attenuation of the efficacy in preclinical models. Agonist antibodies specific to OX40 (anti-OX40) can induce significant antitumor effects in preclinical models. Combination studies performed with anti-PD1 and anti-OX40 demonstrate that concurrent combination treatment induces a strong, but short-lived burst of intratumoral T-cell proliferation, which coincides with acute cytokine secretion, increased TIM-3 + CD8+ exhausted cells, and attenuated antitumor effect. However, administering anti-OX40 and anti-PD-1 in a sequential fashion avoids the T-cell exhaustion induced by concurrent combination treatment [5]. A similar observation was noted in a B-cell lymphoma model, an abrogation of the therapeutic effect of 4-1BB co-stimulation when anti-PD-1 was combined concurrently.

**Fig. 1:**
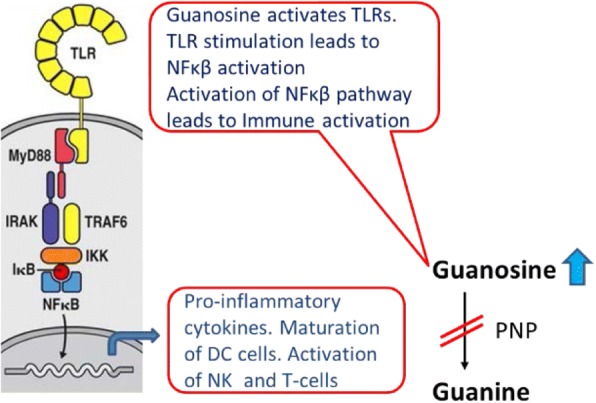
Elevation of guanosine with PNP inhibition causes immune activation through TLRs

A perfect example of overactivation or constitutive activation leading to immune exhaustion can be observed in purine nucleoside phosphorylase (PNP) deficient patients. These patients present with lymphopenia and all along it was thought that inhibition of PNP would lead to immune-suppression and hence PNP inhibitors were developed for autoimmune diseases and hematologic malignancies [6]. Contrary to all prior publications and patents, recently it was discovered that PNP inhibitors are actually immune stimulating agents [7]. Inhibition or deficiency of PNP leads to elevation of guanosine which activates toll like receptors (TLRs). Activation of TLRs is known to stimulate the immune system through NF-κβ pathway (Fig. 1).

PNP deficiency or inhibition causing hyperactivation of the immune system is noted in genetic, preclinical and clinical studies. Examples of this phenomenon include the following (a) autoimmune manifestations, such as lupus, hemolytic anemia and idiopathic thrombocytopenic purpura, are relatively common in PNP deficient patients [6], (b) PNP inhibitor acts as an adjuvant, increasing immune response to tetanus toxoid vaccine both in preclinical and clinical studies [7, 8], and (c) graft-vs-host disease (GVHD), a hallmark of immune activation in Hematopoietic Stem Cell Transplant (HSCT) and noted side effect of immunotherapies (like anti-PD1 and anti-CTLA4) in post-HSCT relapse patients, is common in PNP deficient patients receiving blood transfusion and in post-HSCT relapse patients receiving PNP inhibitor [6, 9]. Lymphopenia noted in PNP deficient patients is primarily related to constitutive activation leading to immune exhaustion.

Another possible clinical example comes from clinical trials of Indoleamine 2,3-dioxygenase − 1 (IDO-1) inhibitor, epacadostat in combination with anti-PD1 immunotherapy. Epacadostat (given daily) in combination with anti-PD1 failed to reach primary endpoint that is Progression free survival (PFS) and overall survival (OS) in the phase III trials of unresectable metastatic melanoma (data presented April 2018) in spite of a number of positive phase I/II trials in melanoma and other solid tumors [10]. While the exact reason for failure in phase III trials is not known, one could speculate that daily administration of the IDO-1 inhibitor could have caused chronic immune activation leading to immune exhaustion.

Chronic/constitutive activation of immune system releasing proinflammatory factors promotes tumor development, progression, and metastatic dissemination. Although, signals that trigger acute inflammatory reactions often stimulate dendritic cell maturation and antigen presentation thus activating antigen specific T-cells and promoting anti-tumor effects. This antagonism between inflammation and immunity needs to be considered carefully.

## Design the clinical trials to define optimum duration of therapy

One could potentially envision immune based therapies to provide the necessary impetus at the beginning and, afterwards, discontinue the therapy further and let the activated immune system control the tumor growth. This concept has been nominally explored in a couple of retrospective or observational studies with the most notable being a follow-up to the KEYNOTE-001 trial for Pembrolizumab [11]. They suggest stopping treatment altogether is a viable option in patients with complete response (CR) as the durability of the response is maintained in about 80–90% of the patients. Although, the motivation and timing for stopping treatment were study duration or Pharmacoeconomics, these results provide initial evidence that treatment duration with immunotherapies cannot be adopted from chemotherapy trials but needs to be rigorously studied [11]. Treatment holidays and possibly stopping immune based therapy early is a concept that needs further research using novel trial designs.

The fear of undertreatment can make clinicians and patients reluctant to pursue abbreviated dosing schedules with immune based therapies. However, with the understanding that over-treatment with immune based treatment could be counterproductive and may potentially promote tumor growth, the issue of treatment duration with immune based therapies need to be seriously addressed. Randomized trials are needed to explore not just stopping early, but also possible predictors of success with an earlier stop. Extensive correlative studies need to be performed with immune-based therapies to identify appropriate biomarkers with clinical response. Because of the complexity of the immune response and tumor biology, it is unlikely that a single biomarker will be sufficient to predict clinical outcomes in response to immune-based therapy. Rather, the integration of multiple tumor and immune response parameters, such as protein expression, genomics, cell subsets, and transcriptomics, may be necessary for accurate prediction of clinical benefit.

In addition to these biomarkers, it may be important to explore immune-related adverse events as potential marker and a possible predictor of success with an earlier termination of therapy. A comparative detailed retrospective analysis of progression free survival and overall survival from patients who received immune based therapies and experienced immune related adverse effects leading to discontinuation of therapy versus patients who continued with therapy may provide valuable information.

As the field for the immune-based therapies is expanding exponentially, the time is now to address the treatment duration for immune based therapies. Overactivation or constitutive activation leading to Immune exhaustion is a real phenomenon and of profound concern as immune cells are the true arsenal for control of the tumor growth. By addressing these concerns now, not only we may improve patient outcomes, but also gather a deeper understanding of the role and mechanisms of the immune system in the control of tumor growth.
