# Correction to: Treatment duration with immune-based therapies in Cancer: an enigma

**DOI:** 10.1186/s40425-018-0486-8

**Published:** 2019-02-26

**Authors:** Shanta Bantia, Nirmal Choradia

**Affiliations:** 1 Nitor Therapeutics, 689, Highland Lakes Cove, Birmingham, AL-35242 USA; 2 Palo Alto Veterans Affairs Hospital, 3801 Miranda Ave, Palo Alto, CA 94304 USA

**Correction to: Journal for ImmunoTherapy of Cancer (2018) 6:143**

**Doi: 10.1186/s40425-018-0465-0**

Following publication of the original article [1], the authors reported an error in the typesetting of their article. The first section of the main text was mistakenly included in the abstract. This section begins with the sentence “Chemotherapy and immune-based therapies provide antitumor effects through completely different mechanisms” and ends with the following sentence:

“In exploring treatment duration with immune based therapies, we need to answer the following: (1) does indefinite treatment with immune based therapies exhaust the immune system counteracting its own mechanism of action leading to tumor progression and (2) how can clinical trials be designed to identify the optimal duration of immunebased therapy that prevents immune cell exhaustion but supports anti-tumor immunity.”

The published apologizes for any inconvenience caused by this error.
